# Hemicircular Tibial Plateau Leveling Osteotomy (hTPLO) for Dogs with Excessive Tibial Plateau Angles: A Comparative Study Using 3D Bone Models

**DOI:** 10.3390/vetsci13070648

**Published:** 2026-07-02

**Authors:** Kyuman Cho

**Affiliations:** 1 Chokyuman Veterinary Surgical Center, Bucheon 14624, Republic of Korea; kyuman@hanmail.net; 2 Veterinary Medical Research Institute, Jeju National University, Jeju 63243, Republic of Korea

**Keywords:** tibial plateau leveling osteotomy, hemicircular TPLO, cranial cruciate ligament rupture, canine orthopedics, excessive tibial plateau angle, surgical technique

## Abstract

A comparative study on hemicircular tibial plateau leveling osteotomy (hTPLO) and TPLO for correcting cranial cruciate ligament (CCL) ruptures at excessive tibial plateau angles (eTPAs) was performed with a specially fabricated hTPLO saw blade (hemicircular) and TPLO saw blade (quartercircular), respectively. Ten 3D-printed bone models with eTPAs (TPA > 40°), were resized to accommodate the respective saw blade shape for each method. Cranial closing wedge osteotomy (CCWO) combined with TPLO was also performed. Distances of segment rotation below the patellar tendon insertion (PTI) and differences between anatomical and mechanical axes after correction were measured. The TPAs of the bone models were 46.06 ± 4.40° (40.0–50.7°). Postoperative TPAs of hTPLO, TPLO, and TPLO combined with CCWO were 4.93 ± 1.025°, 4.52 ± 0.085°, and 4.17 ± 1.128°, respectively. Rotation distances from the PTI were 0.46 ± 2.153 mm, 8.67 ± 2.318 mm, and 1.13 ± 1.796 mm, respectively. hTPLO showed significantly less movement of the segment below the PTI than TPLO with or without CCWO (*p* < 0.01). Differences between anatomical and mechanical axes after hTPLO and TPLO with or without CCWO were 5.74 ± 2.231°, 6.43 ± 2.105°, and 5.16 ± 2.465°, respectively. Within the limitations of this model-based study, hTPLO demonstrated geometric feasibility for correction of excessive tibial plateau angles and was associated with less rotational displacement of the proximal tibial segment relative to the patellar tendon insertion than conventional TPLO and TPLO combined with CCWO. Further cadaveric, biomechanical, and clinical studies are required to evaluate its safety and clinical applicability.

## Simple Summary

This study compared hemicircular tibial plateau leveling osteotomy (hTPLO) with conventional tibial plateau leveling osteotomy (TPLO) for correcting cranial cruciate ligament rupture in dogs with excessive tibial plateau angles. The procedures were performed using a specially fabricated hemicircular saw blade for hTPLO and a quartercircular saw blade for TPLO, respectively. Ten 3D-printed bone models with eTPAs (TPA > 40°), were resized to accommodate the respective saw blade shape for each method. Cranial closing wedge osteotomy (CCWO) combined with TPLO was also performed. Distances of segment rotation below the patellar tendon insertion (PTI) and differences between anatomical and mechanical axes after correction were measured. The TPAs of the bone models were 46.06 ± 4.40° (40.0–50.7°). Postoperative TPAs of hTPLO, TPLO, and TPLO combined with CCWO were 4.93 ± 1.025°, 4.52 ± 0.085°, and 4.17 ± 1.128°, respectively. Rotation distances from the PTI were 0.46 ± 2.153 mm, 8.67 ± 2.318 mm, and 1.13 ± 1.796 mm, respectively. hTPLO showed significantly less movement of the segment below the PTI than TPLO with or without CCWO (*p* < 0.01). Differences between anatomical and mechanical axes after hTPLO and TPLO with or without CCWO were 5.74 ± 2.231°, 6.43 ± 2.105°, and 5.16 ± 2.465°, respectively. In this 3D-printed tibial model study, hTPLO achieved the target postoperative tibial plateau angle and was associated with less rotational displacement of the proximal tibial segment relative to the patellar tendon insertion than conventional TPLO and TPLO combined with CCWO. These findings support the geometric feasibility of hTPLO; however, further cadaveric, biomechanical, and clinical investigations are required before clinical recommendations can be made.

## 1. Introduction

Cranial cruciate ligament (CCL) rupture is one of the common causes of hind limb lameness in dogs [1,2,3]. Various surgical techniques have been developed to correct instability of the stifle joint resulting from a CCL rupture, and among these options, tibial plateau leveling osteotomy (TPLO) is currently the most preferred technique [4,5,6]. The principle of TPLO is to reduce the tibial plateau angle (TPA) to approximately 5° to eliminate cranial tibial thrust instability caused by CCL rupture [7,8,9]. However, performing a leveling osteotomy in dogs with an excessive tibial plateau angle (eTPA, defined as a TPA > 34°), is more challenging [10,11]. Reducing an excessive TPA to 5° with TPLO requires a substantial rotation of the proximal tibial fragment. Rotation below the patellar tendon insertion (PTI) site can cause fractures, typically occurring at the base of the tibial tuberosity and opposite the tibial plateau buttress [12]. This aligns with the tibial tuberosity fracture patterns observed clinically after TPLO surgery [13,14]. Therefore, the magnitude of proximal fragment rotation relative to the patellar tendon insertion (PTI) was selected as a geometric parameter of interest because excessive rotation may increase displacement of the tibial tuberosity segment and potentially contribute to mechanical complications. A combination technique of TPLO and cranial closing wedge osteotomy (CCWO) has been reported to reduce rotation in eTPA [10]. However, when TPLO is combined with CCWO, the surgical procedure becomes more complex, requiring additional implants, and is associated with a higher complication rate compared to TPLO alone.

In this study, we investigated whether complete and safe correction in patients with eTPA is possible using a single TPLO technique. Hemicircular sawing with a diameter of the saw blade within the proximal tibia was hypothesized to be able to rotate the proximal segment of the tibia to the desired TPA, with a shorter distance than the TPLO quartercircular sawing. Providing sufficient space for plate and screw fixation while using the smallest possible diameter for the osteotomy is essential. To satisfy these requirements, a new modified TPLO shape, termed hemicircular tibial plateau leveling osteotomy (hTPLO), has been developed, along with the production of a hemicircular saw suitable for this procedure. Experimental studies using 3D-printed bone models evaluated whether hTPLO could reduce proximal segment displacement relative to the patellar tendon insertion, compared to traditional TPLO, and achieve geometric effects comparable to TPLO combined with CCWO.

## 2. Materials and Methods

### 2.1. Dogs for 3D-Printed Bone Models of the Tibia

**Figure 1:**
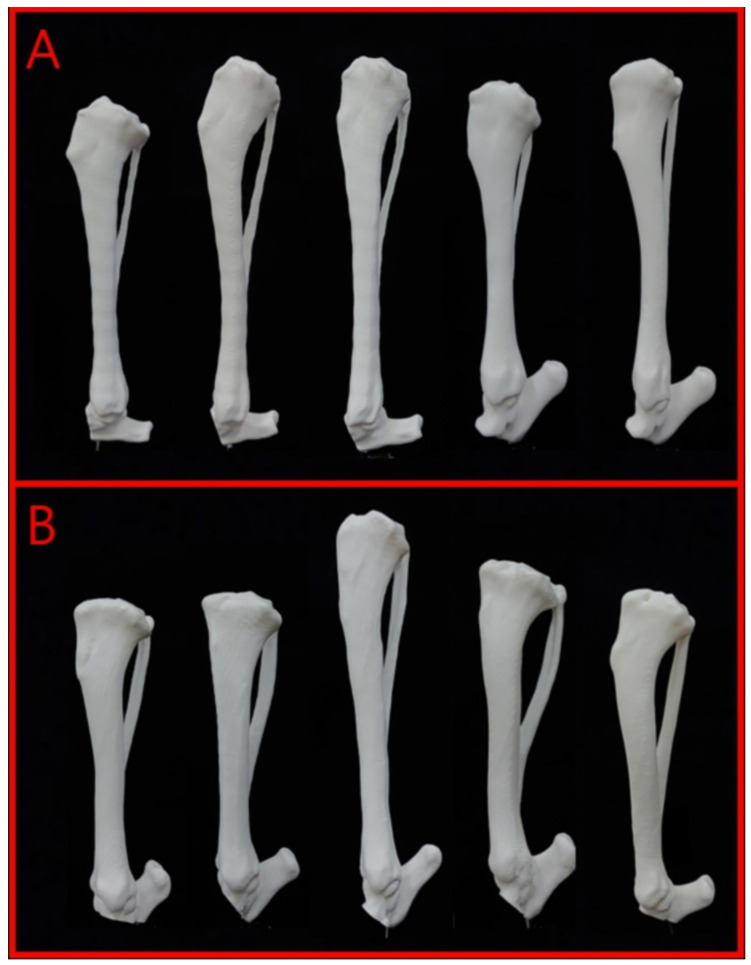
3D-printed bone models of the tibia from patients with cranial cruciate ligament rupture. (**A**) medium and large breed dogs; (**B**) small breed dogs.

Five medium and large breed dogs (>15 kg), and five small breed dogs (of 2.5–15 kg), all with a CCL rupture, were used for 3D printing. TPAs for the large breed dogs were 40.5°, 48.5°, 49.0°, 50.7°, and 45.0° (Figure 1A) and those for the small breed dogs were 40.0°, 40.8°, 50.6°, 45.0°, and 50.5° (Figure 1B), respectively.

### 2.2. 3D Printing

The bone model images of five small breed dogs were created using computed tomography (CT) scans. These CT images were acquired during routine clinical evaluations for diagnostic or surgical planning purposes, and were not obtained specifically for this study. All scans were performed under general anesthesia, induced with propofol (Freepol-MCT^®^, Daewon Pharmaceutical, Seoul, Republic of Korea) and maintained with isoflurane (Terrell™, Piramal Critical Care, Bethlehem, PA, USA). While bone model images of five medium and large breed dogs were created using the free-form deformation (FFD) technique [15,16], with radiographs instead of CT scan images. By adjusting the coordinates of the vertices of the rectangular space using the FFD technique, the object was deformed while maintaining the original shape. This technique allowed modification of the bone template to create a patient-specific bone model using two orthogonal radiographs. Bone models from the images were printed with a 3D printer (CUBICON Style NEO-A31C, CUBICON, Seongnam, Gyeonggi, Republic of Korea) using Cubicreator4 software (CUBICON, Seongnam, Gyeonggi, Republic of Korea) and employing ABS (styrene) filament. The sizes of the bone models were adjusted to accommodate the TPLO saw with a radius of (R)24 mm and the hTPLO saw with R15 mm.

### 2.3. Saws

**Figure 2:**
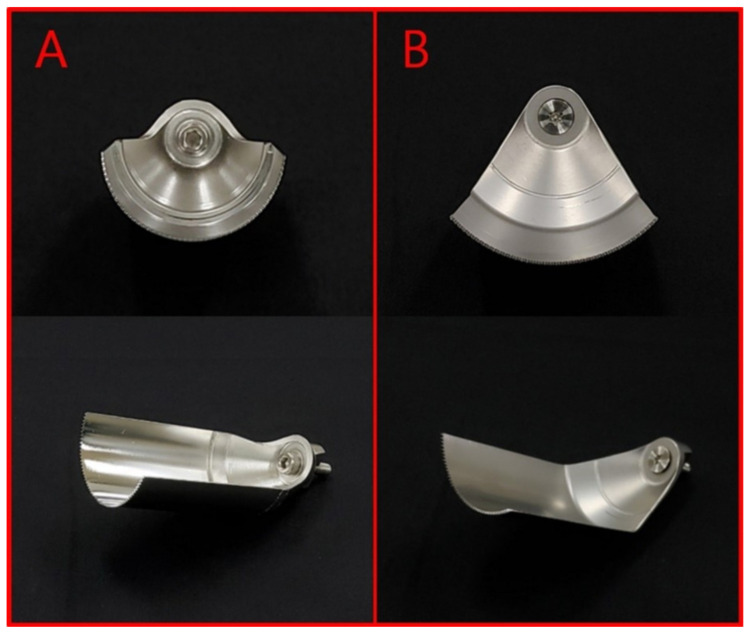
Saw blades. (**A**) Hemicircular tibial plateau leveling osteotomy (hTPLO) saw blade (hemicircular); (**B**) Tibial plateau leveling osteotomy (TPLO) saw blade (quartercircular).

A hemicircular saw was specially fabricated by a local metalworking company (Deokin Precision, Daejeon, Republic of Korea). It was not commercially available and was custom-manufactured using stainless steel 304 via CNC machining and laser welding. The TPLO saw, along with the TPLO plate and screws, was purchased from Synthes (West Chester, PA, USA) (Figure 2).

### 2.4. Surgical Planning

**Figure 3:**
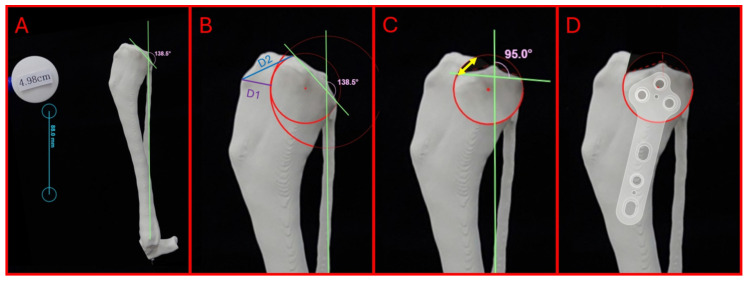
hTPLO surgical design. (**A**) Measurement of size using an indicator and TPA. (**B**) The cranial portion of the hemicircular saw is adjusted to overlap with the D1 position from PTI in the TPLO design. (**C**) Measurement of rotation distance after rotation to the target angle (95°, corresponding to a target postoperative TPA of 5°). (**D**) Plate placement and partial bone removal if necessary.

The osteotomy design was conducted using preoperative orthopedic planning software (vPOP Pro, VetSOS Education Ltd., Llangollen, Wales, UK). TPLO is centered at the intersection of the tibial long axis and the tibial plateau axis (Figure 3A). The tibial long axis was defined as the line connecting the middle of the intertubercular eminences and the center of the tarsal joint, whereas the tibial plateau axis was defined as the line connecting the cranial and caudal margins of the tibial plateau. The osteotomy was planned to leave a tibial tuberosity width of 25–30% of the proximal tibia, with D1 and D2 measured from PTI (Figure 3B). The PTI was defined as the distal insertion point of the patellar tendon on the tibial tuberosity. Rotation distance was defined as the arc distance traveled by the proximal tibial segment from the PTI during rotation to the target postoperative TPA of 5° (Figure 3C). In TPLO combined with CCWO, angles > 34° in the osteotomy design were reduced using CCWO, followed by TPLO. A TPLO saw, sized R24 mm, was used in both methods. In the hTPLO design, the cranial portion of the hemicircular saw is adjusted to overlap with the D1 position in the TPLO design (Figure 3B), using the smallest diameter saw that secures the fixation area of the TPLO plate and screws. Hence, a hTPLO saw, sized R15mm, was used. In the software, the segment gradually rotated until the TPA reached 5° post-osteotomy and the rotation distance was then measured. Each surgical method was performed based on the information obtained from the software. The bone models were secured using a Synthes 3.5 mm TPLO plate, four locking screws, and two cortical screws. In TPLO combined with CCWO surgery, in addition to plate and screws fixation, a tension-band wire (0.4 mm orthopedic wire) and two 1.8 mm K-wires were used for additional fixation. Postoperative photographs were taken using the same method as pre-surgery, and outcomes were measured and evaluated using the software.

For each of the ten tibial geometries, three separate 3D-printed tibial models were produced. One model was assigned to hTPLO, one to conventional TPLO, and one to TPLO combined with CCWO. Therefore, each surgical technique was performed on an identical tibial geometry, allowing direct comparison among techniques while minimizing anatomical variation.

Because the procedures were performed on 3D-printed bone models, a TPLO jig was not used. Osteotomy planning and fragment rotation were performed according to the measurements obtained from the preoperative planning software. In the hTPLO procedure, only the hemicircular osteotomy was performed; a conventional quartercircular TPLO was not additionally created. The geometry of hTPLO differs from that of conventional TPLO because the hemicircular osteotomy results in a different rotational relationship between the proximal and distal tibial segments. As illustrated in Figure 3C, osteotomy geometry incorporated a 95° angle (90° + target TPA of 5°) to achieve the target postoperative tibial plateau angle (TPA) of 5°.

### 2.5. Statistical Analysis

Data are presented with means and standard deviations. Differences among the three surgical techniques were analyzed using repeated-measures one-way analysis of variance (ANOVA) followed by Tukey’s multiple comparisons test. Statistical analyses were performed using GraphPad Prism 8.0.1.244 software. A *p*-value < 0.05 was considered statistically significant.

## 3. Results

**Figure 4:**
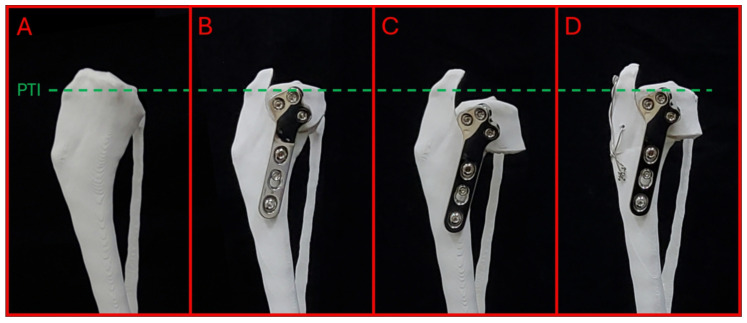
Correction of TPA to 5° for a tibia with eTPA. TPLO technique rotated the proximal segment down and caudally, more than other techniques. (**A**) Tibia with a TPA of 48.5°; (**B**) hTPLO; (**C**) TPLO; (**D**) TPLO + CCWO.

The TPAs of the bone models were 46.1 ± 4.40° (40.0–50.7°). Postoperative TPAs of the hTPLO, TPLO, and TPLO combined with CCWO were 4.93 ± 1.025°, 4.52 ± 0.085°, and 4.17 ± 1.128°, respectively (Table 1). This indicates that all three surgical techniques resulted in consistent postoperative TPA values, demonstrating stable angular changes after surgery. Rotation distances from the PTI were 0.46 ± 2.153 mm, 8.67 ± 2.318 mm, and 1.13 ± 1.796 mm, respectively. The proximal segment in hTPLO exhibited a significantly shorter rotation distance from the PTI than those of the proximal segments in both TPLO and TPLO combined with CCWO (*p* < 0.01). The TPLO technique resulted in a greater rotation distance of the proximal tibial segment below the PTI, compared to the other techniques (Figure 4). Additionally, unlike the other techniques, hTPLO did not cause posterior protrusion of the proximal tibial segment, thus avoiding the balcony effect (Figure 4B). The differences between the anatomical and mechanical axes after hTPLO, TPLO, and TPLO combined with CCWO, were 5.7 ± 2.23°, 6.4 ± 2.11°, and 5.2 ± 2.47°, respectively. The difference between the anatomical and mechanical axes was not statistically significant, suggesting that all surgical techniques used in the experiment resulted in minimal changes in the axes.

## 4. Discussion

In 3D-printed bone models with eTPA, hTPLO using a hemicircular saw was evaluated under the hypothesis that a hemicircular saw could rotate the proximal segment of the tibia to the desired TPA with a shorter distance, as compared to the TPLO quartercircular saw. Results of this study revealed that the rotation distance of the proximal tibial segment in hTPLO surgery for eTPA was significantly less than that in TPLO surgery.

Patients with eTPA require a substantial rotational correction of the segment during TPLO to achieve the target TPA [17,18]. In such cases, the incidence of postoperative complications is higher, with the most common being a loss of postoperative TPA during the recovery period, commonly referred to as rockback [17,19]. The addition of supplementary implants during TPLO significantly reduces the occurrence of this problem. hTPLO may reduce the risk of postoperative rockback and decrease the need for additional implants in eTPA cases because it requires less rotational correction and results in a relatively larger osteotomy contact area than conventional TPLO. These potential advantages are based on the geometric characteristics observed in the present study. However, they were not directly evaluated and require further biomechanical and clinical investigation.

Tibial tuberosity fractures are a frequently encountered complication following TPLO surgery, with reported incidence rates ranging 3.1–7.3% [20,21,22]. However, in large breed dogs with eTPA, the incidence of tibial tuberosity fractures at full rotation of the segment with TPLO is 11%, nearly twice the rate seen with under-rotation [17]. Limiting the rotation of the tibial plateau segment to the PTI to preserve the caudal buttress support of the tibial tuberosity may reduce the incidence of tibial tuberosity fractures; one approach to prevent tibial fractures in cases of eTPA is combining TPLO with CCWO or with other techniques [10,23,24].

In this study, despite achieving the target TPA with hTPLO, the tibial plateau descended significantly less, compared to TPLO surgery. Additionally, the descent below the PTI was less compared to the combined TPLO and CCWO procedure. However, when TPLO is combined with CCWO, applying a larger resection angle during CCWO may alter results, as this significantly affects the anatomical-mechanical angle (AMA) and patella height, requiring careful consideration. Considering these geometric findings, hTPLO resulted in less distal displacement of the proximal tibial segment relative to the PTI. Whether this influences the incidence of tibial tuberosity fractures requires further biomechanical and clinical investigation. Furthermore, the complication rate following combined TPLO and CCWO surgery was reported to be 77.8%, which is significantly higher compared to the complication rate of 18.8–28% when TPLO was performed alone [10]. hTPLO is less invasive than the combined TPLO and CCWO procedure, requiring fewer osteotomies and no additional fixation devices, making it a relatively simpler technique. Postoperative infection following TPLO in dogs weighing >50 kg was closely associated with the stability of the construct [25]. Specifically, in cases where the preoperative TPA > 30°, the “balcony effect” increases the caudal mechanical load, leading to greater micromotion and instability at the osteotomy site [26,27]. This can alter the vascular network and create conditions conducive to bacterial proliferation. However, in eTPA, hTPLO does not induce the balcony effect (Figure 4B). Therefore, differences in postoperative load distribution may exist compared with conventional TPLO. However, micromotion, construct stability, and bacterial proliferation were not evaluated in the present study. The potential effects of hTPLO on osteotomy stability, particularly in larger dogs, require further biomechanical and clinical investigation. Although the present study did not evaluate fracture risk, construct stability, implant loading, or clinical outcomes, hTPLO showed less rotational displacement of the proximal tibial segment relative to the PTI than TPLO and TPLO combined with CCWO. Whether these geometric differences translate into reduced postoperative complications or improved clinical outcomes remains to be determined.

After correction, the AMA was smaller in hTPLO compared to TPLO, and even smaller when TPLO was combined with CCWO. The difference in AMA between hTPLO and TPLO is thought to result from the center of rotation in hTPLO being positioned slightly lower than that of TPLO. Generally, CCWO induces significant changes in the AMA due to considerable movement of the tibia [28]. However, the lack of statistically significant differences in AMA between TPLO combined with CCWO, and either TPLO or hTPLO, may have resulted from the fact that only the segment with an angle > 34° was osteotomized during CCWO.

TPLO and tibial tuberosity transposition (TTT) are often performed concurrently in dogs with CCL rupture and medial patellar luxation (MPL) [29]. Meticulous surgical technique is required to minimize postoperative failure of tibial tuberosity fixation [30]. Excessive rotational displacement of the proximal tibial segment relative to the PTI may complicate procedures involving the tibial tuberosity. In the present study, hTPLO showed significantly less rotational displacement relative to the PTI than conventional TPLO, which may facilitate fixation of the tibial tuberosity when additional procedures such as TTT are required.

hTPLO is considered a promising method for the correction of cases with eTPA, as it involves a smaller radius and shorter rotation distance of the osteotomy segment compared to TPLO. However, because of the smaller osteotomy radius, small rotational errors during hTPLO may result in relatively larger deviations from the target postoperative TPA. Therefore, hTPLO may require greater surgical precision than conventional TPLO. Careful measurement and fixation are necessary to minimize technical errors. Additionally, further biomechanical and clinical studies are required to evaluate the application of hTPLO in dogs with CCL rupture and eTPA. Furthermore, the relatively small number of tibial models evaluated should also be considered a limitation of this preliminary study. In addition, dimensional accuracy, segmentation accuracy, and printing reproducibility of the 3D-printed models were not formally evaluated and therefore represent limitations of the present study.

## 5. Conclusions

The results of this study support the geometric feasibility of hTPLO for the correction of excessive tibial plateau angles.

## Figures and Tables

**Table 1:** Postoperative TPA, rotational distances from the patella tendon insertion, and anatomical and mechanical axes differences between pre and postoperations in relation to surgical techniques.

	hTPLO	TPLO	TPLO + CCWO
Postoperative TPA	4.93 ± 1.025°	4.52 ± 0.085°	4.17 ± 1.128°
Rotational distance (mm)	0.46 ± 2.153 ^a^	8.67 ± 2.318 ^b^	1.13 ± 1.796 ^b^
Anatomical and mechanical axes differences	5.74 ± 2.231°	6.43 ± 2.105°	5.16 ± 2.465°

*n* = 10; ^a^ vs. ^b^, *p* < 0.01. TPA, tibial plateau angle; hTPLO, hemicircular tibial plateau leveling osteotomy; TPLO, tibial plateau leveling osteotomy; CCWO, cranial closing wedge osteotomy.

## Data Availability

The original contributions presented in this study are included in the article. Further inquiries can be directed to the corresponding author.
